# World Health Organization “School Mental Health Manual”-based training for school teachers in Urban Lahore, Pakistan: study protocol for a randomized controlled trial

**DOI:** 10.1186/s13063-018-2679-3

**Published:** 2018-05-24

**Authors:** Nazish Imran, Atif Rahman, Nakhshab Chaudhry, Aftab Asif

**Affiliations:** 1 Child and Family Psychiatry Department, King Edward Medical University/Mayo Hospital, Lahore, Pakistan; 2 University of Liverpool Institute of Psychology, Health and Society, Liverpool, UK; 3 King Edward Medical University/Mayo Hospital, Lahore, Pakistan; 4 Academic Department of Psychiatry and Behavioural Sciences, King Edward Medical University, Lahore, Pakistan

**Keywords:** Children, Schools, Mental health literacy, Teacher, Intervention, Promotion, Socio-emotional skills

## Abstract

**Background:**

The teacher’s role in school mental health initiatives cannot be overemphasized. Despite global evidence of educational interventions in improving teachers’ knowledge and attitudes regarding mental health, this area remains under researched in Pakistan. This paper presents a study protocol of a pilot randomized controlled trial to examine the effectiveness of a teacher training intervention for improving mental health literacy and self-efficacy among school teachers in urban Lahore, Pakistan.

**Methods:**

The randomized controlled trial will follow the CONSORT guidelines. Participants will be allocated to the Intervention group (receiving the World Health Organization, Eastern Mediterranean Region (WHO-EMRO) School Mental Health Manual-based intervention in three 6-h, face-to-face sessions) or a waitlist control group (not receiving training during the study period). Participants will be teachers of private schools with similar broad demographic characteristics in an inner city area of Lahore. The primary outcome measures for the trial is teachers’ mental health literacy. It will be assessed by using the previously applied (during WHO training of Master Trainers) self-administered questionnaire in both groups pre and post training and at 3 months’ follow-up. Secondary outcomes include: for teachers: Teachers’ self-efficacy (assessed by the Teachers’ Sense of Self Efficacy Scale (TSES) short form.); for students (11–16 years): socio-emotional skills and psychological problems measured by the Strengths and Difficulties Questionnaire (assessed at baseline and 3 months post intervention); for schools: the WHO School Psychosocial Profile Questionnaire (baseline and 3 months post intervention).

**Discussion:**

Given the high prevalence of child mental health problems, stigma and lack of services, it is important to consider alternate avenues for promoting positive mental health among youth. This pilot study should establish the effectiveness of the WHO-EMRO School Mental Health Manual-based Intervention improving teacher’s mental health literacy and helping them to learn practical steps that can be implemented at low cost in school settings. It will also provide information regarding intervention implementation and sustainability.

**Trial registration:**

ClinicalTrials.gov, ID: NCT02937714. Registered on 18 October 2016.

**Electronic supplementary material:**

The online version of this article (10.1186/s13063-018-2679-3) contains supplementary material, which is available to authorized users.

## Background

Pakistan is the sixth most populous country of the world with population over 188 million [1]. Almost 35% of population is under the age of 14 years [1]. Child and adolescent mental health thus needs to be considered and emphasized as an integral component of overall health and growth of this young population. The high prevalence of emotional and behavioral problems [2, 3] along with stigma of psychiatric problems and limited child and adolescent mental health services in the country, makes schools a natural platform for mental health promotion. The World Health Organization (WHO) also identifies the promotion of emotional health and well-being as a core feature of their health-promoting school initiative [4]. Schools can play an important role in promoting positive mental health, reducing stigma, raising awareness among teachers, parents and children about mental health issues as well as identifying and supporting youth experiencing mental health difficulties.

Teachers’ role in the school mental health initiatives cannot be overemphasized. Teachers’ own knowledge and beliefs regarding mental health influence the way that they respond to students’ mental health crises [5]. Studies suggest that teachers feel overwhelmed and incompetent to address mental health needs of their student due to their incompetence and lack of knowledge and skills in this area [6, 7]. Teachers’ education is identified as an effective mental health intervention in school settings promoting positive mental health and a sense of connectedness among stakeholders and facilitating student learning, thus improving the overall school atmosphere. Educational interventions involving teachers have shown significant improvement in teachers’ knowledge and attitudes regarding mental health and better accuracy of teacher identification of children and adolescents with mental health problems [8]. Knowledge improvement, reduction of stigma, and confidence in providing support to students as well as indirect positive effects on students were observed in a cluster randomized trial of mental health first-aid training of high school teachers [9]. Furthermore, mental health initiatives in schools help teachers to feel less stressed and be more satisfied with their role, and to save costs [10].

Although the importance of school for mental health promotion and early identification of mental health problems and teachers’ training in mental health has been evident for quite some time and has gained significant momentum in the West, it is almost non-existent in developing countries including Pakistan. Research from Pakistan on school mental health is very limited. In one study done in 1998 in rural Rawalpindi, a school-based programme succeeded in improving mental health awareness in school children, their parents, friends and neighbors; however, teachers’ awareness was not assessed [11]. Knowledge of school teachers regarding attention deficit hyperactivity disorder (ADHD) symptomatology improved in a teacher training programme for ADHD [12]. School mental health initiatives including steps to improve teacher’s mental health literacy, their professional development and training, encouraging teaching styles that promote mutual respect, and positive classroom strategies are almost non-existent in the country. Child mental health is considered a priority in the WHO’s Eastern Mediterranean Region (WHO-EMRO). Recognizing the need for wider implementation of evidence-based school mental health interventions in the region and to help in addressing the mental health literacy of educators in resource-constrained settings, a pilot study of a teachers’ training intervention based on the WHO-EMRO Manual of School Mental Health in Urban Lahore was planned [13]. Training aims for improving teacher’s mental health literacy and helping them to take practical steps that can be implemented at low costs in school settings. The intervention will be evaluated in a pilot randomized controlled trial (RCT), with teachers on a waitlist as the control group, to assess its effectiveness in improving teachers’ mental health literacy and self-efficacy in dealing with students’ mental health issues. It will also enable us to test the feasibility of key aspects of the trial design. The aim of this paper is to describe the study protocol of the pilot RCT.

### Aims and objectives of the study

This pilot RCT seeks to demonstrate the effectiveness of a teacher training programme based on the WHO-EMRO Manual of School Mental Health in improving teacher’s mental health literacy as compared to a waitlist control group. The secondary objective is to evaluate the effect of the WHO-EMRO School Mental Health Manual-based intervention in improving self-efficacy among school teachers. The study will also determine if mental health training of teachers may lead to an indirect improvement in a students’ outcome measure (emotional and behavioral difficulties) and the school psychosocial environment.

## Methods

### Study setting

The study setting is private schools located in urban Lahore, having monthly fee structure of less than 3000 Pakistani rupees and matching in broad characteristics like student:teacher ratio, facilities, etc. Seven schools, from inner city areas of Lahore, are likely to be involved in the study in order to achieve the needed sample size.

### Participants and sample size

All the teachers in the participating schools of grades 1–10 of both genders will be approached. Exclusion criteria included lack of informed consent, teachers who are leaving/not planning to be in the school in 3 months’ time, and teachers who have not been involved in active teaching in last 6 months. Students of 11–16-years old studying in the participating schools whose parents will give informed consent will also be screened.

A sample size of 220 teachers (110 teachers each in the intervention and control groups) is estimated using 90% confidence interval, 5% absolute precision with expected percentage of improvement on mental health literacy in the intervention group as 10% and the control group as 1% [14].

### Study design

**Fig. 1:**
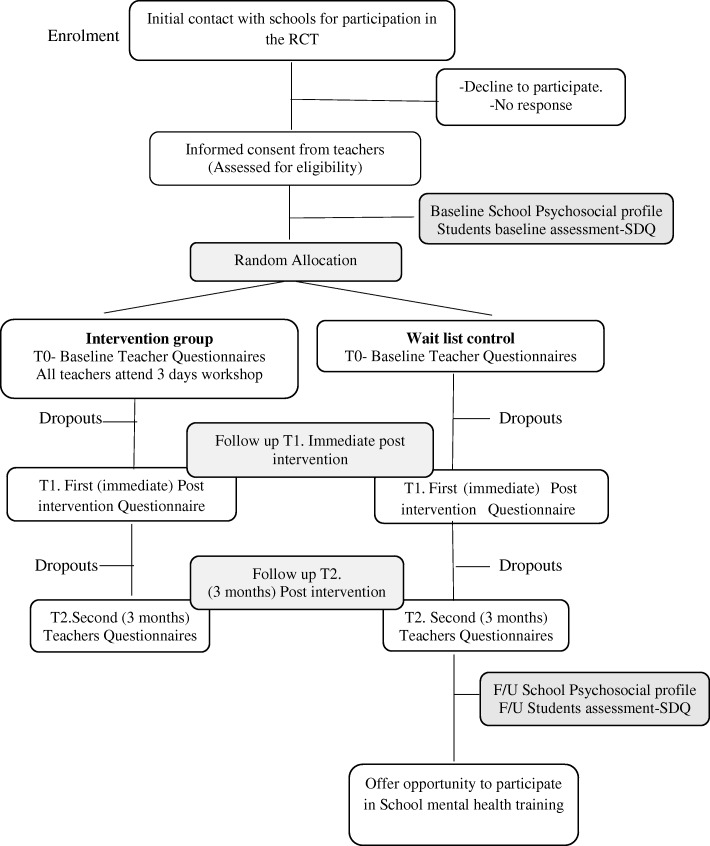
Standard Protocol Items: Recommendations for Interventional Trials (SPIRIT) Diagram

The study is a two-arm RCT. We expect to include five to six schools to obtain the required sample size. Schools will be sent a letter inviting them to participate. All the teachers in the consenting schools, who are willing to participate, will be recruited for the study. Participants will be randomly allocated to the intervention group (receiving the school mental health intervention during the study) or the waitlist control group (teachers who will not receive any training during the study period) using computer-generated random numbers. In order to evaluate the effectiveness of training, a pre- and post-study design will be used (see the Standard Protocol Items: Recommendations for Interventional Trials (SPIRIT) Diagram in Fig. 1). Participants’ mental health literacy and self-efficacy will be evaluated using questionnaires pre and post training and at 3 months’ follow-up. Students (11–16 years old) taught by these teachers and the school psychosocial environment will also be screened at baseline and 3 months after the intervention using the self-reported SDQ. The study follows the Consolidated Standards of Reporting Trials (CONSORT) Guidelines for the design and implementation of RCTs [15].

Additional file 1 shows details of the SPIRIT Checklist items to be addressed in the clinical trial in more detail.

### Intervention

The intervention group will receive a training workshop based on a local adaptation of the WHO-EMRO School Mental Health Manual. The training was adapted in light of in-depth interviews by teachers and school administrators and discussions with relevant stakeholders to make it suitable for the Pakistani school system. The training will be three 6-h sessions delivered face-to-face over a 2-week period. Teachers can decline to participate or withdraw at any stage. Topics to be covered in training workshop are given in Table 1.

**Table 1:** Summary of the modules in the World Health Organization, Eastern Mediterranean Region (WHO-EMRO) School Mental Health Manual

Module	Description
Module 1. Social-Emotional Childhood Development	• Developmental tasks of primary-school-age children (6–12 years) • Developmental tasks of secondary-school-age children (12–18 years) • Moral development • Brain development and implications for schooling
Module 2. Mental Health Promoting Schools (Promotion and Prevention)	• Core values of a mental health promoting school • The role of parents in their children’s education • Behavioral management strategies for schools Discipline and management of disruptive behavior Counseling Circle time • Physical health promoting efforts that impact mental health Nutrition Vision/ hearing/ speech Physical exercise • Media and mental health Screen time Internet addiction Cyberbullying • Suicide prevention
Module 3. Addressing Student Mental Health Problems in Your Classroom (and when to refer for additional help)	• Recognizing warning signs for different disorders: Depression Anxiety Pervasive developmental disorders Attention disorders Psychotic disorders Specific learning disorders Intellectual disability Conduct disorders Substance abuse • When to refer to a specialist for evaluation and treatment

The workshop will be conducted by the principal investigator (PI) along with one to two assistants (psychologists trained by the PI). The PI was trained over a period of 2 days by one of the WHO trainers and a Master Trainer of school mental health. (NA and PA). Participants will be provided with standardized school mental health manual handouts.

### Procedure for informed consent

Schools directors/heads will be approached and a letter will also be sent inviting them to participate. All the teachers in the consenting schools who are willing to participate will be recruited for the study. Teachers will be invited to attend an informal meeting/workshop by the PI, who will provide an overall view of the study. A description of the WHO-EMRO Manual including its rationale, the format of the interventions proposed and anticipated potential benefit to the target population will be provided. The workshop will also help to gain key stakeholder support for the project and augment understanding of local schools mental health strategies. Teachers will also have the opportunity to discuss and clarify any aspect of the project. Written informed consent will be obtained from teachers before participating in the RCT. To ensure anonymity, participating teachers will be requested not to provide any identifying information on the test questionnaire, rather to anonymously identify information like mother’s first name. This information will be used to link pre-and post-training responses. All the parents of eligible students (11–16 years) in the participating schools will receive an information letter regarding the teacher training, study project and data collection of their children. and a written informed consent form. Assent from the students will be required prior to answering the questionnaire.

### Randomization

Participants will be randomly allocated to the intervention group (receiving school mental health intervention during the study) or the waitlist control group (no training) using computer-generated random numbers. Randomization will be done by a statistician not directly linked to the study using the teachers list provided by the schools. The intervention delivery team will not be involved in the randomization procedure.

#### Blinding

**Fig. 2:**
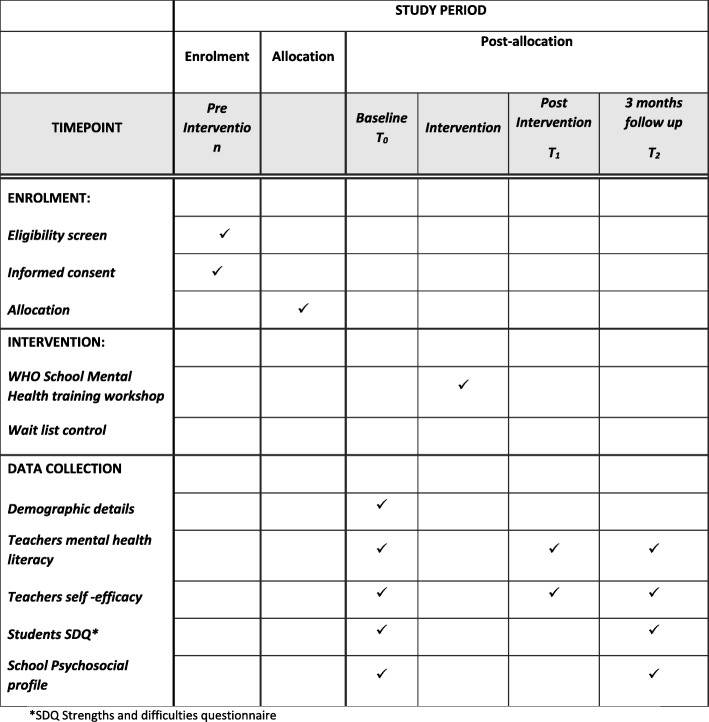
Trial schema

It is not possible for study participants to be blind to intervention status. However, other staff members assisting with data collection from teachers and students, data input and the statistical support persons helping with analysis will be blind to the intervention status (Fig. 2).

### Outcome measures

Table 2 shows the outcomes to be collected at each time point.

**Table 2:** Outcomes to be collected by time point

	Baseline	Immediate post intervention	3 months’ follow up
Teacher	Mental health literacy: Self-administered Knowledge, Skills and Attitudes Questionnaire^a^ Teachers’ Sense of Self Efficacy Scale (TSES): short form	Mental health literacy: Self-administered Knowledge, Skills and Attitudes Questionnaire^a^ Teachers’ Sense of Self Efficacy Scale (TSES): short form	Mental health literacy: Self-administered Knowledge, Skills and Attitudes Questionnaire^a^ Teachers’ Sense of Self Efficacy Scale (TSES): short form
Students	Strengths and Difficulties Questionnaire (SDQ) 11–16	–	Strengths and Difficulties Questionnaire (SDQ) 11–16
Schools	Psychosocial Profile Questionnaire	–	Psychosocial Profile Questionnaire

^a^Primary outcome

#### Primary outcome: assessment of mental health literacy of teachers, using a self-administered questionnaire

The primary outcome measures for the trial is teachers’ mental health literacy immediately post intervention. Mental health literacy refers to a person’s knowledge and beliefs about mental disorders, which in turn increases their ability to recognize, manage, or prevent the development or exacerbation of mental health problems [16]. It has various elements including: (1) enhancing the capacity to obtain and maintain good mental health; (2) enhancing understanding of mental disorders and their treatments; (3) decreasing stigma related to mental illness; and (4) enhancing help-seeking efficacy [17].

It will be assessed by using previously applied (during WHO training of Master Trainers) pre and post tests in both groups, that were reviewed for cultural appropriateness by Pakistani mental health experts. The initial section of questionnaire has 30 multiple choice questions. Possible responses are ‘true’, ‘false’ and ‘I don’t know’. The next section has six stories with five questions each of possible interventions to be considered by school teachers. Each question has a ‘yes’, ‘no’ format. Participants will be instructed to choose only one option per question and will be encouraged to mark ‘I don’t know’ rather than guessing. A total score out of 60 maximum will be calculated.

Demographic information, length of teaching experience, information about classes they teach (primary, secondary, both), and previous training on similar topics will also be collected from all teachers in both groups.

#### Secondary outcome measures

##### Teachers

*Teachers’ Sense of Self Efficacy Scale (TSES): short form*

The TSES measures teachers’ assessment of their self-efficacy for teaching tasks as well as their other responsibilities [18]. There are 12 statements and each statement is rated on 9-point Likert scale. Permission was obtained from the authors of the scale. Teachers’ efficacy in student engagement and classroom management subscales will be used in the study, as they relate to the content of the intervention.

##### Students

*Strengths and Difficulties Questionnaire (SDQ)*

The Strengths and Difficulties Questionnaire (SDQ), a universally validated tool, will be used to screen students for behavioral and emotional problems. The SDQ has various versions; parents, teachers and self-administered for students. We will be using, self- reported SDQ (11–16 years). The SDQ measures 25 attributes, which are grouped in subscales of five items each, generating scores for conduct, hyperactivity, emotional, peer problems and prosocial behavior. Statements are rated on a 3-point Likert scale:0 (not true), 1 (somewhat true) and 2 (certainly true). All scales excluding the last are added to generate a total difficulties score (0–40). Total difficulties and categories scores can be coded into normal, borderline and abnormal categories [19, 20].

##### Schools

*School Psychosocial Profile Questionnaire*

The WHO Psychosocial Profile Questionnaire has grouped various school characteristics in seven quality areas. An average score of each quality area is calculated.

### Statistical analysis

Data will be analyzed using SPSS 20. A description of baseline characteristics of schools and of individuals who participate in the two intervention arms will be compiled using descriptive statistics such as mean and standard deviation, frequency and percentages. Aggregates of the mean item score will be created for each scale at three timelines (baseline T0, immediately following intervention T1, and 3 months following intervention T2). Paired samples’ *t* tests will be used to examine differences in knowledge and attitudes scores and self-efficacy between participants at T0, T1 and T2. Differences in mean scores between the study and control groups will be evaluated using an independent samples’ *t* test. Intention-to-treat analysis will be used for data analysis. Student outcome measures for schools and the psychosocial environment will be compared at baseline and follow-up using the *t* test. Statistical significance will be fixed at a level of *P* < .05. Data on screening, refusals and dropouts will be reported as per Consolidated Standards of Reporting trials (CONSORT) guidelines for participant flow through the trial.

### Ethical considerations

The Institutional Review Board of King Edward Medical University has approved the study protocol. The trial has also been registered at the ClinicalTrials.gov registry (NCT02937714). Information will be provided to teachers across the schools as well as students to ensure that they are aware of an agreed referral process within the school, should they identify a need for psychological assessment or treatment.

#### Data monitoring

The study does not have any risks of harm. However, the PI will provide oversight to the trial and any concerns expressed by any participants during the study will be documented and discussed within the team and supervisor to consider appropriate solutions. The PI and the supervisors will have access to the datasets.

### Feedback of the training

Participants will be asked to provide quantitative and qualitative feedback regarding the content, delivery and quality of training at the end of workshop by using a feedback questionnaire. This feedback will be used to further adapt and refine the training for the larger RCT.

### Dissemination

A report will be prepared for each participating school containing the overall findings and also, from each school, anonymised results from the teacher and student questionnaire. Papers reporting the outcomes will be published in peer-reviewed journals. If the findings show the intervention to be effective, it will help in refining the large-scale RCT and the results will be disseminated to policy-makers in the private and Government sectors for this training to be included in mandatory teacher training.

## Discussion

Despite evidence of more than half of all mental disorders having an onset in childhood and adolescence [21] and poor mental health in young age being associated with school failure, delinquency, social and peer problems, substance misuse alongside adverse outcomes in adulthood, child mental health is not given due consideration and importance in countries with a high proportion of young people. The WHO report highlights that ‘Lack of attention to the mental health of children and adolescents may lead to mental disorders with lifelong consequences, undermines compliance with health regimens and reduces the capacity of societies to be safe and productive’ [22].

Low-income countries, like Pakistan, face a multitude of social adversities including poverty, malnutrition, rapid urbanization, educational deprivation, drug abuse, increased crime, terrorism, etc., thus increasing the risk of mental health problems in youth. However, there has been very limited child mental health epidemiological research in the country of the unmet need for child mental health services and the effectiveness of evidence-based interventions. Given the high prevalence and lack of services, it is important to consider alternate methods for promoting the psychological and emotional well-being of our youth. In this scenario, schools can act as an important platform for promoting positive mental health in children and adolescents in Pakistan.

The aim of this paper is to describe the protocol of a RCT regarding an intervention programme designed to improve teachers’ knowledge, attitude and skills to promote children’s socio-emotional well-being and mental health in the school context in Pakistan. The presented intervention is based on the WHO-EMRO School Mental Health Manual. Specifically, the aim of this pilot RCT is to provide more information on adapting evidence methods for diverse cultures and related school settings. We are not aware of any previous RCT done in Pakistan focusing on teacher training in a whole-school approach. A particular strength of the study is its focus on secondary student’s outcome as well. The limited time available for the study makes the follow-up time of 3 months more feasible rather than 6 months, which might have been more appropriate to observe any significant change. Another limitation of the trial is the generalizability of the results to the Government sector and to elite private schools, which differ significantly in various socio-demographic variables and will not be targeted in this pilot trial. Contamination between teachers in the intervention and control groups may occur when recruiting teachers within the same school and is a limitation of the trial. Findings including the effectiveness and implications from this study will provide information supporting the large-scale RCT for training teachers to increase their mental health literacy and thus potentially improving future school mental health services. In addition, the trial will provide important information about any implementation issues and the extent to which it is likely to be sustainable in different settings in the country. Positive trends result from this trial will hopefully support the urgent work needed in school mental health in Pakistan.

## Trial status

Recruitment of schools for the study started in December 2016. The trial is currently underway.

## Additional file

Additional file 1: Standard Protocol Items: Recommendations for Interventional Trials (SPIRIT) 2013 Checklist: recommended items to address in a clinical trial protocol and related documents. SPIRIT Checklist with details of page numbers in which recommended items in a clinical trial are addressed. (DOC 119 kb)

## Abbreviations

RCT: Randomized controlled trial
SDQ: Strengths and difficulties Questionnaire
TSES: Teachers’ Sense of Self Efficacy Scale
WHO-EMRO: World Health Organization, Eastern Mediterranean Region

## References

[1] World Population Prospects: The 2015 Revision, Key Findings and Advance Tables. United Nations, Department of Economic and Social Affairs, Population Division. 2015. Working Paper No. ESA/P/WP.241.

[2] Syed E, Hussein SA, Mahmud S (2007). Screening for emotional and behavioural problems amongst 5–11-year-old school children in Karachi, Pakistan. Soc Psychiatry Psychiatr Epidemiol.

[3] Syed E, Hussein SA, Haidry SZ (2009). Prevalence of emotional and behavioural problems among primary school children in Karachi, Pakistan—multi informant survey. Indian J Pediatr.

[4] World Health Organization (1998). WHO’s Global School Health Initiative: Health Promoting Schools..

[5] Graham A, Phelps R, Maddison C, Fitzgerald R (2011). Supporting children’s mental health in schools: teachers views. Teach Teach Theory Pract.

[6] Froese-Germain B, Riel R. Understanding teachers’ perspectives on student mental health: Findings from a national survey. Ottawa: Canadian Teachers’ Federation; 2012. Retrieved from https://www.ctf-fce.ca/Research-library/StudentMentalHealthReport.pdf.

[7] Whitley S, Smith JD, Vaillancourt T (2013). Promoting mental health literacy among educators: critical in school-based prevention and intervention. Can J Sch Psychol.

[8] Kutcher S, Wei Y, McLuckie A, Bullock L (2013). Educator mental health literacy: a programme evaluation of the teacher training education on the mental health and high school curriculum guide. Adv School Ment Health Promot.

[9] Jorm AF, Kitchener BA, Sawyer MG, Scales H, Cvetkovski S (2010). Mental health first aid training for high school teachers: a cluster randomized trial. BMC Psychiatry.

[10] Weist MD, Murray M (2007). Advancing school mental health promotion globally. Adv School Ment Health Promot.

[11] Rahman A, Mubbashar MH, Gater R, Goldberg D (1998). Randomised trial of impact of school mental-health programme in rural Rawalpindi, Pakistan. Lancet.

[12] Syed EU, Hussein SA (2010). Increase in teachers’ knowledge about ADHD after a week-long training program: a pilot study. J Atten Disord.

[13] Manual of School Mental Health. World Health Organization, Eastern Mediterranean Regional Office; 2014.

[14] Jorm AF, Kitchener BA, Sawyer MG, Scales H, Cvetkovski S (2010). Mental health first aid training for high school teachers: a cluster randomized trial. BMC Psychiatry.

[15] Kenneth FS, Douglas GA, David M (2010). CONSORT 2010 Statement: updated guidelines for reporting parallel group randomised trials. BMJ.

[16] Jorm AF (2012). Mental health literacy. Empowering the community to take action for better mental health. Am Psychol.

[17] Kutcher S, Wei Y (2014). School mental health literacy: a national curriculum guide shows promising results. Educ Can.

[18] Tschannen-Moran M, Woolfolk Hoy A (2001). Teacher efficacy: capturing and elusive construct. Teach Teach Educ.

[19] Goodman R (1999). Psychometric properties of the Strengths and Difficulties Questionnaire. J Am Acad Child Adolesc Psychiatry.

[20] Samad L, Hollis C, Prince M, Goodman R (2005). Child and adolescent psychopathology in a developing country: testing the validity of the Strengths and Difficulties Questionnaire (Urdu version). Int J Methods Psychiatr Res.

[21] Kessler RC, Amminger GP, Aguilar-Gaxiola S, Alonso J, Lee S, Ustun TB (2007). Age of onset of mental disorders: a review of recent literature. Curr Opin Psychiatry.

[22] World Health Organization (2003). Caring for Children and Adolescents with Mental Disorders: Setting WHO Directions.

